# Novel synthetic (E)-2-methoxy-4-(3-(4-methoxyphenyl) prop-1-en-1-yl) phenol inhibits arthritis by targeting signal transducer and activator of transcription 3

**DOI:** 10.1038/srep36852

**Published:** 2016-11-15

**Authors:** Dong Ju Son, Dae Hwan Kim, Seong-Su Nah, Mi Hee Park, Hee Pom Lee, Sang Bae Han, Udumula Venkatareddy, Benjamin Gann, Kevin Rodriguez, Scott R. Burt, Young Wan Ham, Yu Yeon Jung, Jin Tae Hong

**Affiliations:** 1 College of Pharmacy and Medical Research Center, Chungbuk National University, Cheongju, Chungbuk 28160, Korea; 2 Division of Rheumatology, Department of Internal Medicine, College of Medicine, Soonchunhyang University, Asan, Chungnam 31538, Korea; 3 Department of Chemistry and Biochemistry, Brigham Young University, Provo, UT 84604, USA; 4 Department of Chemistry, Utah Valley University, 800 W University Pkwy, Orem, UT 84058, USA; 5 Department of Dental Hygiene, Gwangyang Health Sciences University, Gwnagyang, Jeonnam 57764, Korea

## Abstract

Rheumatoid arthritis (RA) is a severely debilitating chronic autoimmune disease that leads to long-term joint damage. Signal transducer and activator of transcription 3 (STAT3)-targeted small molecules have shown promise as therapeutic drugs for treating RA. We previously identified (E)-2,4-bis(p-hydroxyphenyl)-2-butenal (BHPB), a tyrosine-fructose Maillard reaction product, as a small molecule with potent anti-inflammatory and anti-arthritic properties, mediated through the inhibition of STAT3 activation. The aim of this study was to develop a novel BHPH derivative with improved anti-arthritic properties and drug-likeness. We designed and synthesised (E)-2-methoxy-4-(3-(4-methoxyphenyl) prop-1-en-1-yl) phenol (MMPP), a novel synthetic BHPB analogue, and investigated its anti-inflammatory and anti-arthritic activities in experimentally-induced RA. We showed that MMPP strongly inhibited pro-inflammatory responses by inhibiting *in vitro* STAT3 activation and its downstream signalling in murine macrophages and human synoviocytes from patients with RA. Furthermore, we demonstrated that MMPP exhibited potent anti-arthritic activity in a collagen antibody-induced arthritis (CAIA) mouse model *in vivo*. Collectively, our results suggest that MMPP has great potential for use in the treatment of RA.

Rheumatoid arthritis (RA) is a highly debilitating chronic autoimmune disease that leads to long-term joint damage, and often results in chronic pain, swelling, stiffness, loss of physical function, and reduced life expectancy. RA is characterised by synovial hyperplasia and inflammatory cell recruitment, and intra-articular fibrin deposition, as well as cartilage and bone destruction^1^,^2^. Despite recent advancements in the pharmacological treatment of RA, the lack of response to current pharmaceutical drugs in a proportion of patients, as well as therapy discontinuation due to drug side effect, remain unsolved significant limitations^3^. Therefore, new strategies that actively target RA are required, and higher efficacy therapeutic drugs are urgently needed to treat RA.

Signal transducer and activator of transcription 3 (STAT3) is a potentially important transcription factor in a number of autoimmune diseases^4^. In RA, STAT3 is a well-known key mediator of chronic joint inflammation, synovial fibroblast proliferation, and joint destruction, and constitutive activation of STAT3 in synoviocytes and lymphocytes is implicated in patients with RA^5^,^6^,^7^,^8^. Therefore, STAT3 has been proposed as a potential therapeutic target to prevent or treat chronic joint inflammation and RA. Currently, a broad spectrum of therapeutic strategies targeting STAT3 signalling are under development and have shown promising results in preclinical and clinical trials^9^. For example, tofacitinib (an oral Janus kinase 3 [JAK3] tyrosine kinase inhibitor, Pfizer Inc.), which inhibits STAT3 phosphorylation, was approved by the US Food and Drug Administration (FDA) for the treatment of patients with RA who are unresponsive to disease-modifying antirheumatic drug (DMARD) therapy^10^. Further, recent studies showed that the non-peptide small molecules, STA-21 (STAT3 inhibitor) and AG490 (JAK2-STAT3 pathway inhibitor) improve the treatment efficacy considerably in active RA^11^,^12^. Therefore, pharmaceutical approaches and advancements in STAT3-targeted small molecules have emerged as promising pharmacological therapies for RA.

Previously, we also showed that (E)-2,4-bis(*p*-hydroxyphenyl)-2-butenal (BHPB), a bioactive compound derived from a tyrosine-fructose Maillard reaction (MR) product^13^, effectively inhibits the activation of STAT3 and its downstream signalling pathways by directly binding to its DNA-binding domain. This action mediates the potent therapeutic effects of BHPB against RA^14^ as well as Alzheimer’s disease^15^,^16^,^17^, and tumour growth^18^,^19^,^20^,^21^,^22^,^23^. However, its drug-likeness is not relevant for drug development because it has a low chemical stability, low solubility, and high toxicity. Therefore, we sought to develop a novel and improved anti-arthritic agent with applicable drug-likeness properties by designing and synthesized a novel BHPB derivative, (E)-2-methoxy-4-(3-(4-methoxyphenyl) prop-1-en-1-yl) phenol (MMPP). Here, we demonstrated that MMPP strongly attenuates STAT3 activation, which in turn regulates pro-inflammatory factors. Furthermore, MMPP exhibited improved anti-arthritic efficacy in a collagen antibody-induced RA mouse model, with adequate drug-likeness properties.

## Results

### Improved anti-inflammatory activity of MMPP

**Figure 1:**
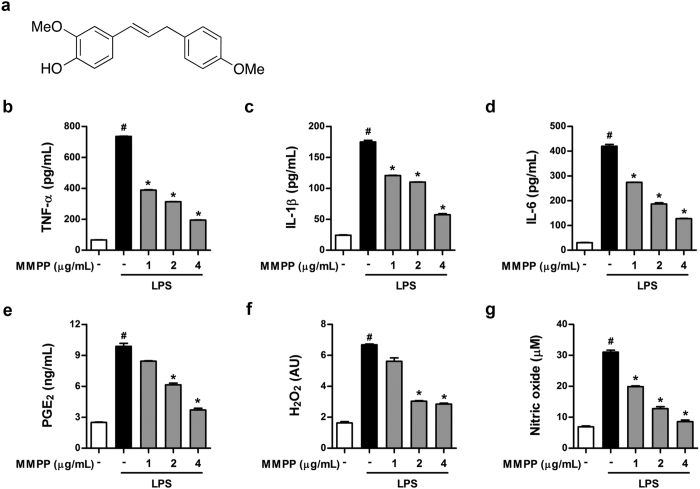
(E)-2-Methoxy-4-(3-(4-methoxyphenyl) prop-1-en-1-yl) phenol (MMPP) inhibits production of pro-inflammatory mediators in LPS-stimulated RAW 264.7 cells. Chemical structure of MMPP shown in (**a**). (**b**–**g**) Effect of MMPP on production of pro-inflammatory mediators in lipopolysaccharide (LPS)-stimulated murine macrophages was determined. RAW 264.7 cells were pre-treated with MMPP (1, 2, and 4 μg/mL) for 24 h, then stimulated with LPS (1 μg/mL) for 24 h. Levels of (**b**) tumour necrosis factor (TNF)-α (n = 5), (**c**) interleukin (IL)-1β (n = 5), (**d**) IL-6 (n = 5), (**e**) prostaglandin E_2_ (PGE_2_, n = 5), (**f**) hydrogen peroxide (H_2_O_2_, n = 8), and (**g**) nitric oxide (NO, n = 8) were determined in culture medium as described in Materials and Methods. All data are mean ± standard error of the mean (SEM). ^#^*P* < 0.05 *vs.* intact control, and ^*^P < 0.05 *vs.* LPS-treated control as determined using Student’s *t*-test.

We initially evaluated the anti-inflammatory activity of newly synthesized BHPB analogues, and compared it with BHPB in RAW264.7 cells, which are a common murine macrophage-like cell line. Among tested 16 compounds, MMPP (Fig. 1a) showed most potent inhibitory effect on the STAT3 transcriptional activity and nitric oxide (NO) generation in lipopolysaccharide (LPS)-stimulated RAW264.7 cells with half-maximal inhibitory concentration (IC_50_) values of 2.05 and 1.79 μg/mL (equivalent to 7.56 and 6.60 μM), respectively. These IC_50_ values were stronger than those of BHPB (5.91 and 6.41 μg/mL equivalent to 23.26 and 25.23 μM) or comparative compounds (curcumin, 4.01 and 5.21 μg/mL equivalent to 10.89 and 14.14 μM; tofacitinib, 4.12 and 5.53 μg/mL equivalent to 13.18 and 17.70 μM) that are currently used clinically for RA treatment targeting STAT3 activation (Supplementary Table S1). Further, we found that the LPS-induced production of pro-inflammatory mediators including tumour necrosis factor (TNF)-α, interleukin (IL)-1β, IL-6, prostaglandin E_2_ (PGE_2_), hydrogen peroxide (H_2_O_2_), and NO (Fig. 1b–g, respectively) in RAW264.7 cells was effectively suppressed by MMPP treatment in a concentration-dependent manner, while the cell viability was not affected (Supplementary Fig. S1a). In addition, TNF-α-induced NO and H_2_O_2_ production was also considerably suppressed by MMPP treatment in RAW264.7 cells (Supplementary Fig. S2a). Together, these results demonstrate that MMPP exhibited a more potent, and improved anti-inflammatory activity than BHPB.

### MMPP potently suppresses STAT3 signalling pathways in murine macrophages

**Figure 2:**
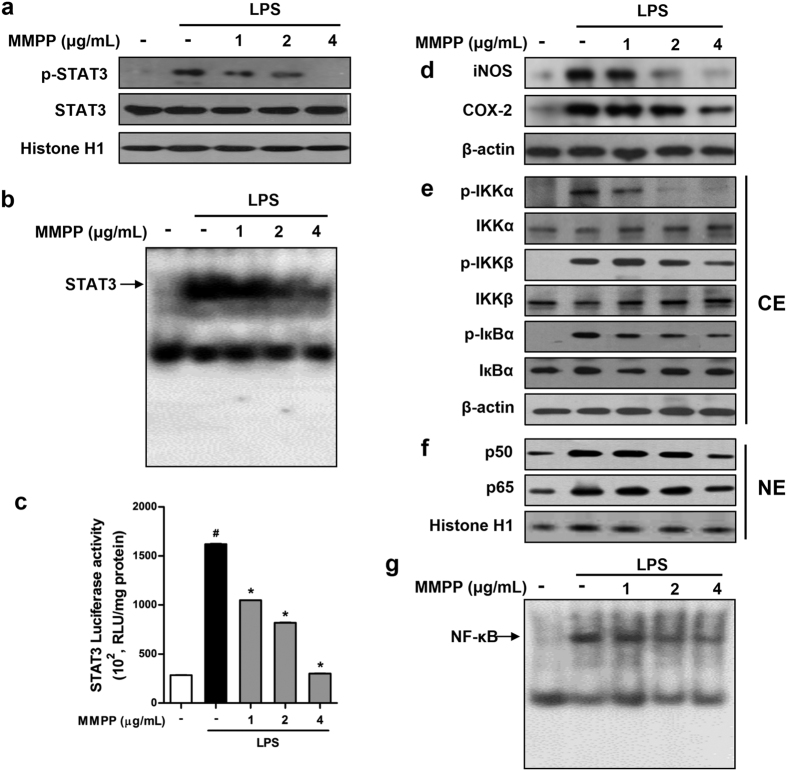
(E)-2-methoxy-4-(3-(4-methoxyphenyl) prop-1-en-1-yl) phenol (MMPP) suppresses signal transducer and activator of transcription 3 (STAT3) and nuclear factor kappa-light-chain-enhancer of activated B cells (NF-κB) activation and pro-inflammatory protein expression in lipopolysaccharide (LPS)-stimulated RAW 264.7 cells. (**a**–**g**) Murine macrophage-like RAW264.7 cells were pre-treated with MMPP (1, 2, and 4 μg/mL) for 24 h, then stimulated with LPS (1 μg/mL) for 24 h. (**a**) Cells were lysed, nuclear fraction was isolated, and then expression of phosphorylated-STAT3 (p-STAT3) and total-STAT3 was analysed using western blot with histone H1 as loading control. (**b**) DNA-binding activity of STAT3 was determined using electrophoretic mobility shift assay (EMSA) with nuclear extracts. (**c**) Effect of MMPP on STAT3 transcription activity was determined using Luciferase reporter gene assay (n = 6, data are mean ± standard error of the mean (SEM), ^#^*P* < 0.05 *vs.* control and ^*^P < 0.05 *vs.* LPS-treated control using Student’s *t*-test). (**d**–**f**) Effect of MMPP on LPS-induced (**d**) iNOS and COX-2 expression, (**e**) IKKα, IKKβ, and IкBα in the cytosol (CE; cytosol extract), (**f**) NF-κB p50 and p65 expression in nuclear (NE: nuclear extract) are shown. (**g**) NF-κB DNA binding activity was detected by EMSA. ^#^*P* < 0.05 *vs.* control, and ^*^P < 0.05 *vs.* LPS-treated control as determined by Student’s *t*-test. iNOS, inducible nitric oxide synthase; COX, cyclooxygenase; IKK, inhibitor of nuclear factor kappa-B kinase; IкB, nuclear factor of kappa light polypeptide gene enhancer in B-cells inhibitor.

To confirm whether the anti-inflammatory effect of MMPP was mediated by its actions on STAT3 activation, we examined STAT3 protein activation change by detecting phosphorylated STAT3 levels in LPS-stimulated RAW264.7 cells. We found that MMPP treatment strongly decreased LPS-induced STAT3 phosphorylation in a concentration-dependent manner (Fig 2a). We further found that MMPP treatment effectively decreased LPS-induced DNA-binding activity of STAT3 in a concentration-dependent manner (Fig. 2b). In addition, the STAT3 luciferase report assay results showed that MMPP treatment considerably inhibited STAT3 transcriptional activity in LPS-stimulated RAW264.7 cells (Fig. 2c). We also confirmed that MMPP inhibited TNF-α-stimulated STAT3 DNA-binding activation (Supplementary Fig. S2b), suggesting that it attenuates STAT3 activation by both LPS and other stimuli. Next, we determined whether MMPP successfully inhibits the pro-inflammatory proteins that are activated by STAT3 signalling and produce inflammatory mediators. MMPP treatment decreased the expression of inducible NO synthase (iNOS) and COX-2 induced by LPS (Fig. 2d) as well as TNF-α (Supplementary Fig. S2c).

The interaction of STAT3 and nuclear factor kappa-light-chain-enhancer of activated B cells (NF-κB) augments the inflammation responses as major regulators of pro-inflammatory signalling pathways^24^,^25^ and, therefore, we investigated the effects of MMPP on the NF-κB pathway. We found that MMPP treatment attenuated LPS-induced phosphorylation of inhibitor of nuclear factor kappa-B kinase (IKK)-α and IKKβ, two catalytic subunits of IKK, and nuclear factor of kappa light polypeptide gene enhancer in B-cells inhibitor (IкB)-α in the cytosol (Fig. 2e). In addition, it also decreased the nuclear translocation of NF-κB subunits (Fig. 2f). In agreement with these results, MMPP treatment decreased the LPS- and TNF-α-induced DNA-binding activity of NF-κB in murine macrophages (Fig. 2g and Supplementary Fig. S2d). These results suggest that the MMPP-induced inhibition of NF-κB signalling under our experimental condition may be mediated by its interference with the interaction between STAT3 and NF-κB signalling pathways.

### MMPP suppresses inflammatory responses in synoviocytes from RA patients

**Figure 3:**
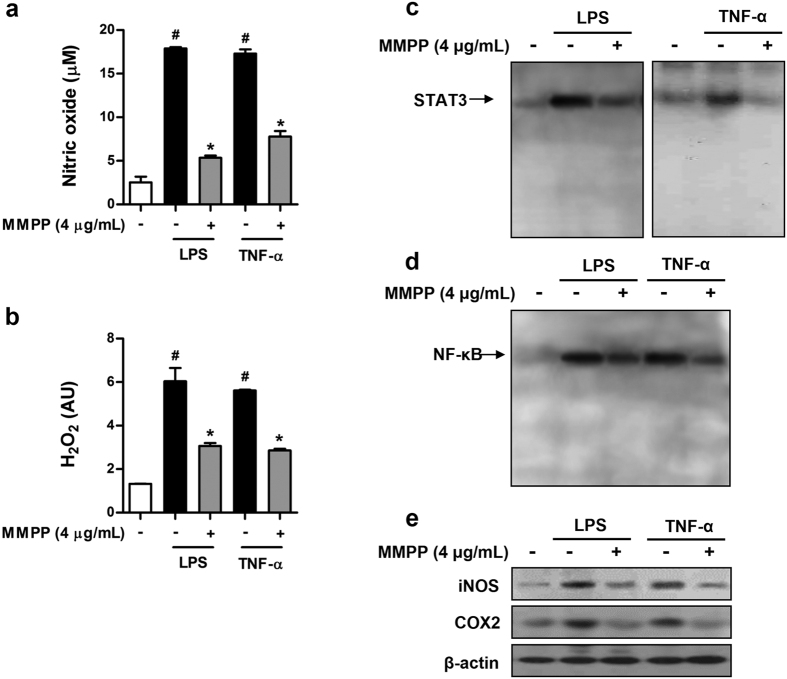
(E)-2-Methoxy-4-(3-(4-methoxyphenyl) prop-1-en-1-yl) phenol (MMPP) inhibits reactive oxygen species (ROS) generation, signal transducer and activator of transcription 3 (STAT3), and nuclear factor kappa-light-chain-enhancer of activated B cells (NF-κB) activation, and pro-inflammatory protein expression in synoviocytes of patients with rheumatoid arthritis (RA). (**a**–**e**) Human synoviocytes isolated from patients with RA were pre-treated with MMPP (4 μg/mL) for 24 h, and then stimulated with LPS (1 μg/mL) or TNF-α (10 ng/mL) for 24 h. Levels of (**a**) nitric oxide (NO) and (**b**) hydrogen peroxide (H_2_O_2_) were determined (n = 6, data are mean ± standard error of the (SEM), ^#^*P* < 0.05 *vs.* intact control and ^*^P < 0.05 *vs.* LPS- or TNF-α-treated control using Student’s *t*-test). (**c**–**d)** DNA-binding activity of (**c**) STAT3 and (**d**) NF-κB was determined using electrophoretic mobility shift assay (EMSA) with nuclear extracts. (**e**) Cells were lysed and analysed using western blot with antibodies against iNOS and COX2 with β-actin as loading control. iNOS, inducible nitric oxide synthase; COX, cyclooxygenase.

To determine the relevance of the anti-inflammatory effect of MMPP in human joint inflammation, we investigated its anti-inflammatory efficacy in human fibroblast-like synoviocytes from patients with long-standing RA. First, we observed that MMPP treatment did not affect synoviocytes viability (Supplementary Fig. S1b). We found that the production of NO (Fig. 3a) and H_2_O_2_ (Fig. 3b) was increased by treating synoviocytes with LPS and TNF-α, and this effect was inhibited by MMPP treatment. We also discovered that the treatment of synoviocytes with MMPP decreased the DNA-binding activity of STAT3 (Fig. 3c) and NF-κB (Fig. 3d) induced by LPS and TNF-α. In addition, the expression of pro-inflammatory proteins, iNOS, and COX-2 was inhibited by MMPP treatment in LPS- and TNF-α-stimulated synoviocytes (Fig. 3e). These results demonstrate that similar to its effects in murine macrophages, MMPP potently inhibited pro-inflammatory responses in human synoviocytes by the same action mechanism that involves STAT3 and NF-κB signalling inactivation.

### Treatment with MMPP reduces CAIA in mice

**Figure 4:**
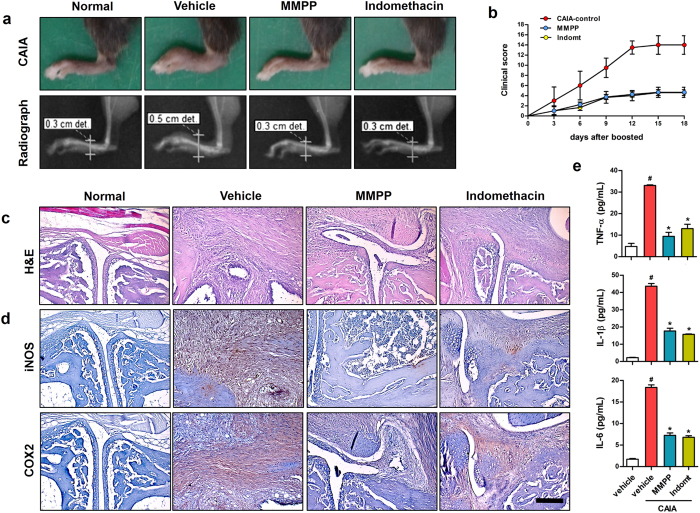
(E)-2-Methoxy-4-(3-(4-methoxyphenyl) prop-1-en-1-yl) phenol (MMPP) treatment reduces rheumatoid arthritis (RA) symptoms in collagen antibody-induced arthritis (CAIA) mouse model. For the assessment of RA, C57BL/6 mice were injected intravenously with anti-collagen II monoclonal antibody on day 0, then challenged with 50 μg of lipopolysaccharide (LPS) intraperitoneally (i.p.) on day 3. Mice were treated i.p. with 0.05% dimethyl sulphoxide (DMSO) in normal saline (vehicle control), 5 mg/kg MMPP, or 5 mg/kg indomethacin (Indomt) daily during entire experimental period. (**a**) On day 21, severity of RA in hind paws was examined using photo-imaging and radiographic analysis. A representative image of each experimental group is shown. Clinical scoring for 18 days after LPS booster injection is shown in (**b)** (*n* = 10 each, data are mean ± standard error of the mean (SEM)). (**c**–**e**) Paraffin sections prepared from ankle joint tissue were stained with (**c**) haematoxylin and eosin (H&E), (**d**) iNOS or COX2. Images are representative microscope images (n = 5). Nuclei (blue) and protein expression (brown) are shown. Scale bar, 100 μm. (**e**) Levels of TNF-α, IL-1β, and IL-6 in ankle joints tissue of mice were determined (n = 5, mean ± SEM, ^#^*P* < 0.05 *vs.* vehicle-treated normal control and ^*^P < 0.05 *vs.* vehicle-treated CAIA-control using Student’s *t*-test). iNOS, inducible nitric oxide synthase; COX, cyclooxygenase; TNF, tumour necrosis factor; IL, interleukin.

**Figure 5:**
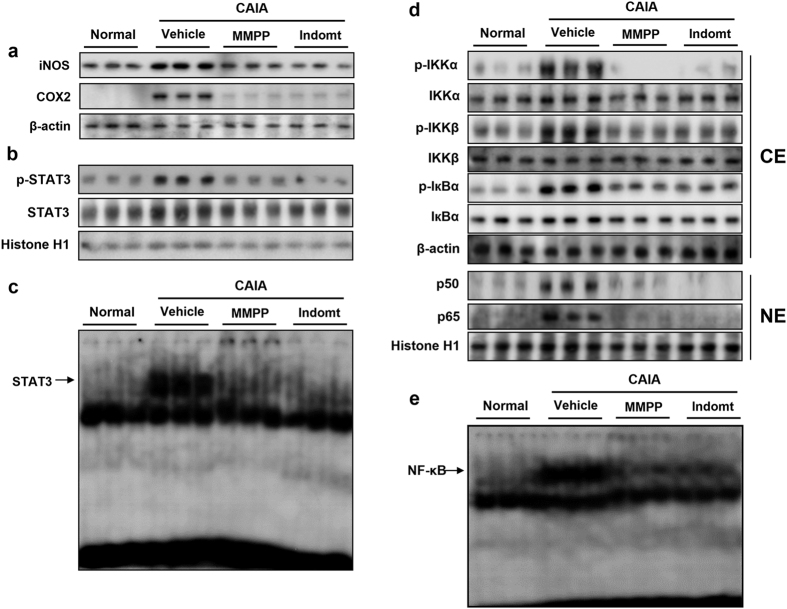
(E)-2-Methoxy-4-(3-(4-methoxyphenyl) prop-1-en-1-yl) phenol (MMPP) treatment inhibits pro-inflammatory protein expression, signal transducer and activator of transcription 3 (STAT3), and nuclear factor kappa-light-chain-enhancer of activated B cells (NF- κB) activation in collagen antibody-induced arthritis (CAIA) mouse model. (**a**–**e**) C57BL/6 mice were injected intravenously with anti-collagen II monoclonal antibody on day 0, and then challenged with 50 μg of lipopolysaccharide (LPS) intraperitoneally (i.p.) on day 3. Mice were treated i.p. with 0.05% dimethyl sulphoxide (DMSO) in normal saline (vehicle control), 5 mg/kg MMPP, or 5 mg/kg indomethacin (Indomt) daily during entire experimental period. On day 21, ankle joints were dissected, and total proteins were extracted and fractionated into cytosolic (CE) and nuclear (NE) fraction (n = 3 each group). Expression of (**a**) iNOS, COX-2, (**b**) phosphorylated-STAT3 (p-STAT3), and total-STAT3 was analysed using western blot. DNA-binding activity of STAT3 was determined using electrophoretic mobility shift assay (EMSA) with the nuclear fraction. Expression of (**d**) IKKα, IKKβ, and IкBα in CE, and (**f**) NF-κB p50 and p65 expression in NE are shown. (**e**) DNA-binding activity of NF-κB in tissues was also determined using EMSA. iNOS, inducible nitric oxide synthase; COX, cyclooxygenase; IKK, inhibitor of nuclear factor kappa-B kinase; IкB, nuclear factor of kappa light polypeptide gene enhancer in B-cells inhibitor.

Finally, we examined the pharmacological role of MMPP in a CAIA mouse model that is currently widely used to study RA^26^. In this study, C57BL/6 mice were treated with intraperitoneal (i.p.) injections of MMPP (5 mg/kg), indomethacin (positive control, 5 mg/kg) or vehicle daily for 21 days. In the CAIA model, as expected, the collagen antibody injection along with a booster (LPS) shot rapidly induced a pronounced RA phenotype including swelling and bone destruction in the ankle and paws, which was blunted by treatment with indomethacin, a clinically used medicine for chronic inflammatory arthritis (Fig. 4a). Notably, treatment with MMPP markedly inhibited RA compared with the vehicle-treated CAIA control group (Fig. 4a). These results were accompanied by a decrease in the clinical score in MMPP- and indomethacin-treated mice, which showed a decrease of 66.5% (clinical score, 4.67) and 64.8% (clinical score, 4.91), compared to that of the CAIA control group (clinical score, 14.15, Fig. 4b). The histopathology in the ankle joints of the CAIA control mice was characterised by an invasion of pannus tissue into the bone space, destruction of the joint architecture, and fibrosis. We found that MMPP treatment dramatically reduced the bone destruction and fibrosis in the CAIA mouse model (Fig. 4c). The increased expression of iNOS and COX-2, which was localized primarily in the fibrous tissue structures surrounding the ankle joint with RA tissue lesions, was markedly reduced by MMPP treatment (Figs 4d and 5a). Furthermore, we found that the elevated pro-inflammatory cytokine (TNF-α, IL-β, and IL-6) levels in the ankle joint tissue were more remarkably reduced in the MMPP-treated mice than they were in the vehicle-treated control mice (Fig. 4e). In addition, we confirmed that the phosphorylation and DNA-binding activity of STAT3 was highly increased in the ankle joint tissues of the CAIA control mice and markedly decreased by the administration of MMPP (Fig. 5b,c). Moreover, suppressed NF-κB signal activation and NF-κB DNA-binding activity were observed in the MMPP-treated mouse ankle joint tissues (Fig. 5d,e).

Considering the importance of immune cells in RA, we examined whether MMPP affects the blood immune cell populations, which is commonly used in diagnosis and progress monitoring of RA. We observed that the number of circulating neutrophils and monocytes in the CAIA control mice blood was markedly elevated compared with that of the normal mice, and this effect was reduced in the MMPP-treated mice (Supplementary Fig. S3a). In addition, the elevated NO level in splenic T-lymphocytes from the CAIA control mice was considerably decreased in the MMPP- and indomethacin-treated groups (Supplementary Fig. S3b). Collectively, these findings demonstrate that MMPP had a potent inhibitory activity against RA by the inhibition of inflammatory responses through the suppression of activation of STAT3 and its downstream signalling pathway.

## Discussion

The identification of STAT3 as a potential therapeutic target for RA has encouraged the development of STAT3-targeted drugs^9^. Small molecules targeting STAT3 have been demonstrated as potential pharmaceutical drugs for chronic joint inflammation and RA^9^,^10^,^11^,^12^. Previously, a DNA-binding site in the STAT3 core fragment was identified as a critical position for regulating STAT3 activity by small molecules^27^,^28^. The present study investigated the effects of MMPP, a novel synthetic STAT3-targeted small molecule BHPB derivative, on inflammation and RA using a CAIA mouse model.

It is well known that MR products exhibit potent anti-inflammatory and antioxidant activities^29^,^30^,^31^,^32^. We previously identified BHPB, a tyrosine-fructose MR product as a STAT3 inhibitor, which has potential therapeutic properties against multiple diseases including RA^15^,^16^,^17^,^18^,^19^,^20^,^21^,^22^,^23^,^33^. Despite its multiple pharmacological properties, the major drawback of BHPB in drug development is its lack of drug-likeness properties and limited chemical stability. To improve its chemical stability, drug-likeness, and pharmacological efficacy, we conducted a major chemical manipulation based on structure-activity relationship (SAR) studies using BHPB as a starting compound. In this study, we designed a group of BHPB derivatives targeting STAT3. Among the candidate compounds, we found that MMPP had the most promising inhibitory effects against STAT3 transcriptional activity. Further, we found that MMPP effectively inhibited the generation of pro-inflammatory mediators (NO, ROS, and PGE_2_ and cytokines), downregulated iNOS and COX-2 expression, and inhibited STAT3 activation. In addition, MMPP inhibited NF-κB signalling in the LPS- and TNF-α-stimulated murine macrophages and human synoviocytes.

We confirmed the anti-inflammatory activity of MMPP, which is mediated by interference with STAT3 activation and its downstream signalling pathways *in vitro*, and we evaluated the therapeutic effects of MMPP on RA *in vivo*. In the present study, we used a CAIA mouse model, which is a widely used animal model of RA, to examine the effects of MMPP treatment on RA progression. Notably, treatment with MMPP dramatically inhibited RA symptoms such as paw swelling, fibrosis, and bone erosion in the ankle joints and improved the clinical score. In addition, our histopathological and blood biochemical examination results supported the potent inhibitory effect of MMPP on pro-inflammatory mediator production, pro-inflammatory protein expression, and STAT3 activation in ankle joint tissues and were consistent with the *in vitro* findings. We also showed that MMPP treatment significantly reduced the circulating neutrophils and monocytes as well as splenic lymphocyte NO production, indicating the beneficial systemic anti-inflammatory effects of MMPP administration.

In the characteristic features of RA, NF-κB activation is well recognized as another pivotal regulator of inflammation along with STAT3. Several reports have suggested that STAT3 could interact with NF-κB, and this interaction between the two transcriptional factors could aggravate the inflammatory responses mediated by pro-inflammatory signalling pathways in numerous inflammatory diseases including RA^24^,^25^,^34^. In the synovium of patients with active RA, concomitant activation of the STAT3 and NF-κB pathways induces a variety of genes that contribute to the inflammatory response such as those for *iNOS* and *IL-6*, which recruit immune cells to the inflamed pannus^35^,^36^. In this study, we showed that treatment with MMPP attenuated the pro-inflammatory responses, which was accompanied by the simultaneous inhibition of NF-κB and STAT3 DNA-binding activities in the ankle joint tissues of the CAIA mice. Taken together, the anti-RA effects of MMPP may be closely related to simultaneous down-regulation of the STAT3 and NF-κB signalling pathways.

Patients with RA usually require lifelong treatment with drugs that can cause adverse effects^37^,^38^. For example, methotrexate has been linked to the development of fatty liver disease and fibrosis during long-term therapy^39^,^40^, and sulphasalazine is well known to cause marrow suppression, nephrotoxicity, hepatotoxicity, and pulmonary toxicity^41^. The hepatotoxicity of leflunomide, an immunosuppressive DMARD used in severe RA was also reported^42^. Therefore, the toxicological and safety evaluation of drug candidates is essential to ensure the development of safe and effective therapeutic drugs. To investigate whether MMPP has favourable drug-likeness properties to be developed as a STAT3 targeted therapeutic medicine for RA, we conducted *in silico* analysis of toxicity and ADME. Our analysis predicted MMPP to be low toxic compound with suitable drug-likeness properties.

In conclusion, we demonstrated that MMPP is an anti-inflammatory compound that strongly inhibits the pro-inflammatory gene and mediator expression and production, respectively, by suppressing STAT3 activation and its downstream signalling pathway in human synoviocytes from patients with RA and murine macrophages. Further, we demonstrated that MMPP exhibited great potential for use in the treatment of RA, with improved drug-likeness. Therefore, MMPP might have the potential for further development as an effective and safe therapeutic agent for treating RA. Further studies are warranted to investigate how this compound can be developed for use in RA therapy.

## Methods

The detailed methods are available in the Supplementary Information.

### Preparation and characterisation of MMPP

We designed and synthesised a library of BHPB analogues with a modification in the conjugated α,β-unsaturated aldehyde moiety, protection of their phenolic alcohols against various ethers, or both. As anticipated, reduction of the alkene or aldehyde of the α,β-unsaturated aldehyde moiety as well as the protection of the phenolic alcohol against ether stabilised the compound since no degradation or polymerization was observed in the thin layer chromatography (TLC) analysis. The compound was designed and prepared to possess a *trans* conformation in the main molecular frame of the alkene without the aldehyde functional group. The compounds were prepared using Heck reaction in a one-step process, successfully obtained at a reasonable yield (25–40%), and purified to homogeneity using flash silica gel column chromatography. The proton nuclear magnetic resonance (^1^H-NMR) characteristics were as follows: (500 MHz, CDCl_3_) δ 7.32 (d, 2H, *J* = 8.0 Hz), 6.88 (d, 1H, *J* = 9.0 Hz), 6.86 (d, 2H, *J* = 9.0 Hz), 6.76 (d, 1H, *J* = 8.0 Hz), 6.75 (s, 1H), 6.40 (d, 1H, *J* = 16.0 Hz), 6.21 (dt, 1H, *J* = 16.0 Hz, *J* = 6.5 Hz), 5.54 (s, 1H), 3.89 (s, 3H), 3.82 (s, 3H), 3.48 (d, 2H, 7.0 Hz). HRMS (ESI) *m/z* [M + H]^+^ cacld. 271.1329, found 271.1332. The MMPP structure is shown in Fig. 1A.

### RAW264.7 Cell culture

The murine macrophage-like cell line RAW 264.7 was obtained from the American Type Culture Collection (ATCC, Manassas, VA, USA), and cultured as previously described^43^. In brief, the cells were cultured in Dulbecco’s modified Eagle’s medium (DMEM) with 10% heat-inactivated foetal bovine serum (FBS) and penicillin/streptomycin (100 U/mL) at 37 °C under a humidified atmosphere containing 5% CO_2_, inside a CO_2_ incubator.

### Human synoviocytes culture and ethics statement

Patients with RA were diagnosed according to the 1987 Revised Criteria of the American College of Rheumatology. Synovial tissue samples were obtained from female and male patients (two each) with long-standing RA [age, 65 ± 21.3 years (mean ± SD); mean disease duration ≥10 years] at the time of a total knee joint replacement. Prior written and informed consent was obtained from each patient, and the study was approved by the Soonchunhyang University Medical Center Ethical Committee. The human synovial tissue sampling and use of human primary cells were performed in accordance with the guidelines approved by the Clinical Research Ethics Committee of Soonchunhyang University College of Medicine. The human fibroblast-like synoviocytes (FLSs) were cultured as previously described^43^. In brief, the FLSs were propagated in culture dishes (Nalge Nunc International, Rochester, NY, USA) in DMEM supplemented with 20% heat-inactivated FBS and penicillin/streptomycin (50 U/mL) at 37 °C under a humidified atmosphere containing 5% CO_2_ inside a CO_2_ incubator. The medium was changed every 3 days, and the cells were used between the fifth and tenth passages.

### Animals and ethics statement

Male C57BL/6 mice (7-weeks-old) were obtained from Taconic Korea (Daehan Biolink Co., Ltd., Umsung, Chungbuk, Korea). The animals were housed under specific pathogen-free conditions with three to four animals per cage on dust-free plant fibre bedding and maintained at 23 ± 2 °C with a controlled 12-h light/dark cycle. The animals were provided drinking water and a rodent chow diet *ad libitum* throughout the experiment. All the animal experiments were conducted in accordance with the principles and procedures outlined in the Korean National Institute of Health Guide for the Care and Use of Laboratory Animals. The protocol for the animal procedures was approved by the Chungbuk National University Institutional Animal Care and Use Committee and compiled with the Korean National Institute of Health Guide for the Care and Use of Laboratory Animals (CBNUA-754-14-01).

### CAIA induction and dosing

To induce CAIA, the 7-week-old male C57BL/6 mice (Daehan Biolink) were given 5 mg collagen II antibody cocktail (CII-Ab, Chondrex, Redmond, WA, USA) intravenously on day 0, challenged with 50 μg (i.p.) on day 3, and were examined visually daily for the appearance of arthritis in the peripheral joints until day 21 (Supplementary Fig. S4). The mice were treated i.p. with vehicle (0.05% dimethyl sulphoxide [DMSO] in normal saline), MMPP (5 mg/kg), or indomethacin (5 mg/kg, a positive control), daily from day 0 to the end of the experiment.

### Histological and CBC tests

The animals were sacrificed, their paw tissues were collected, and histological analyses were performed as previously described^33^. The whole blood samples were corrected for the complete blood count (CBC). The absolute neutrophils and monocytes were counted using a high-volume haematology analyser (Siemens AG, Henkestrasse, Erlangen, Germany).

### Cell viability assay

The cells were treated with MMPP for 24 h, and then 0.2% trypan blue was added to the cell suspension. Subsequently, a drop of suspension was placed in a Neubauer chamber, and the living cells were counted.

### NO and H_2_O_2_ measurements

The cells were stimulated with or without LPS (1 μg/mL) and TNF-α (10 ng/mL) in the absence or presence of various concentrations of MMPP for 24 h. The NO and H_2_O_2_ production in the culture medium was measured using colorimetric (iNtRON Biotechnology, Sungnam, Korea) and fluorometric (Cell Biolabs, San Diego, CA, USA) assay kits, respectively, according to the manufacturer’s instructions.

### PGE_2_ and cytokine measurements

The levels of PGE_2_, TNF-α, IL-1β, and IL-6 were measured using enzyme-linked immunosorbent assay (ELISA) kits (R&D Systems, Minneapolis, MN, USA) according to manufacturer’s instructions.

### Reporter gene assay

The cells were transiently transfected with the STAT3-luciferase reporter (Affymetrix, Santa Clara, CA, USA) or phosphorylated-NF-κB-luciferase reporter (Stratagene, San Diego, CA, USA) using the Lipofectamine LTX and PLUS (Invitrogen, Carlsbad, CA, USA) according to manufacturer’s instruction. The transfected cells were treated with LPS (1 μg/mL) in the absence or presence of MMPP. The reporter gene activity was assayed using the Luciferase assay kit (Promega, Madison, WI, USA).

### Western blot analysis

The whole cell lysates, as well as cytosolic and nuclear extracts of the cultured cells and ankle joint tissues were obtained, separated using sodium dodecyl sulphate-polyacrylamide gel electrophoresis (SDS-PAGE), and the western blot analysis was performed as described previously^33^.

### DNA-binding activity assay using electromobility shift assay (EMSA)

The DNA-binding activities of STAT3 and NF-κB were determined using an electromobility shift assay (EMSA) as described previously^33^.

### Statistical analysis

Statistical analysis was performed using the Graph-Pad Prism 5.0 program (GraphPad Software, La Jolla, CA, USA). Pairwise comparisons were performed using one-way Student’s *t*-tests. The data are presented as means ± standard error of the mean (SEM) of the indicated number of experiments. Differences between groups were considered significant at *P*-values < 0.05.

## Additional Information

**How to cite this article**: Son, D. J. *et al*. Novel synthetic (E)-2-methoxy-4-(3-(4-methoxyphenyl) prop-1-en-1-yl) phenol inhibits arthritis by targeting signal transducer and activator of transcription 3. *Sci. Rep.* **6**, 36852; doi: 10.1038/srep36852 (2016).

**Publisher’s note:** Springer Nature remains neutral with regard to jurisdictional claims in published maps and institutional affiliations.

## Supplementary Material

Supplementary Information
