# Supplementary material for: Novel synthetic (E)-2-methoxy-4-(3-(4-methoxyphenyl) prop-1-en-1-yl) phenol inhibits arthritis by targeting signal transducer and activator of transcription 3
Source: Sci Rep. 2016 Nov 15;6:36852. doi: 10.1038/srep36852 (PMC5109275; doi:10.1038/srep36852)
Supplement: Supplementary Information [file srep36852-s1.pdf]

## Supplementary Information for

# Novel synthetic (E)-2-methoxy-4-(3-(4-methoxyphenyl) prop-1-en-1-yl) phenol inhibits arthritis by targeting signal transducer and activator of transcription 3

Dong Ju Son[1,#], Dae Hwan Kim[1,#], Seong-Su Nah[2], Mi Hee Park[1], Hee Pom Lee[1], Sang Bae Han[1], Udumula Venkatareddy[3], Benjamin Gann[3], Kevin Rodriguez[4], Scott R. Burt[3], Young Wan Ham[4], Yu Yeon Jung[5,*], and Jin Tae Hong[1,*]

[1]College of Pharmacy and Medical Research Center, Chungbuk National University, Cheongju, Chungbuk 28160, Korea; [2]Division of Rheumatology, Department of Internal Medicine, College of Medicine, Soonchunhyang University, Asan, Chungnam 31538, Korea; [3]Department of Chemistry and Biochemistry, Brigham Young University, Provo, UT 84604, USA; [4]Department of Chemistry, Utah Valley University, 800 W University Pkwy, Orem, UT 84058, USA; [5]Department of Dental Hygiene, Gwang Yang Health College, Gwnagyang, Jeonnam 57764, Korea

[#] These authors contributed equally to this work.

[*]**Correspondence should be addressed to**: Jin Tae Hong, PhD, Professor/Director, College of Pharmacy & Medical Research Center, Chungbuk National University, 194-31 Osongsaengmyeong 1-ro, Osong-Biocampus Building Room 303, Osong-eup, Heungdeok-gu, Cheongju, Chungbuk 28160, Korea; Tel.: 82-43-261-2813; Fax: 82-43-268-2732; E-mail: jinthong@chungbuk.ac.kr
or Yu Yeon Jung, PhD, Assistant Professor, Department of Dental Hygiene, Gwang Yang Health College, 85 Daehak-ro, Gwangyang-eup, Gwangyang, Jeonnam 57764, Korea; Tel.: 82-61-760-1486; E-mail: hygienkh@naver.com

## Supplementary Methods

### Synthesis and characterization of (E)-2,4-bis(p-hydroxyphenyl)-2-butenal (BHPB)

Previously, we identified (E)-2,4-bis(p-hydroxyphenyl)-2-butenal (BHPB) as a strong small molecule that has multiple biological activities including anti-arthritic properties by inhibiting signal transducer and activator of transcription 3 (STAT3) activation[15-23,33]. However, we later found that BHPB has low stability due to the highly conjugated α,β-unsaturated aldehyde moiety in its chemical structure, which causes its easy degradation and polymerization even at ambient temperatures. In an effort to improve the drug-likeness properties and biological efficacies, we designed and synthesized a library of 16 BHPB analogues with modifications of the conjugated α,β-unsaturated aldehyde moiety, protection of their phenolic alcohols against various ethers, or both. As anticipated, the reduction of the alkene or the aldehyde of α,β-unsaturated aldehyde moiety resulted in stable compounds. The stability was enhanced by the protection of the phenolic alcohols against ether (compounds **1** – **10**) as evidenced by the lack of degradation and polymerization in TLC analysis. Compounds **8** – **10** were prepared as racemic mixtures and tested directly without separating the two enantiomeric from the mixture. In addition, compounds **11** – **16** were prepared to possess *trans* conformation in the main molecular frame of the alkene and without the aldehyde functional group. These compounds were prepared using a Heck reaction in a one-step process. Compounds **13** and **16** were successfully obtained at a reasonable yield (25–40%) and purified to homogeneity using flash silica gel column chromatography. However, four compounds (**11**, **12**, **14**, and **15**) were often readily isomerized, to our surprise, even at room temperature in the presence of a limiting amount of base in the reaction. They were tested as isomeric mixtures without further purification as a proof of concept because the isomers were extremely difficult to separate.

Compound **1**: Palladium(II) acetate (Pd[OAc]$_2$, 208 mg, 0.93 mmol), tributyl phosphine (685 μL, 2.78 mmol) in t-butanol was degassed under argon gas for 10 min until the solution became clear pale yellow, 4-(oxiran-2-yl) phenyl acetate (5.5 g, 30.87 mmol) was added, and the reaction mixture was stirred at 85°C for 12 h. After completing the reaction, the solvent was removed in *vacuo,* followed by column purification (3:1, hexane:ethyl acetate) to yielded a pale yellow liquid (2 g, 45%). Proton nuclear magnetic resonance ($^1$H NMR, 500 MHz, $CDCl_3$) δ. 9.66 (s, 1H), 7.29 (d, 2H, *J* = 14.5Hz), 7.2 (dd, 4H, *J* = 3.5, 14.5Hz), 7.07 (d, 2H, *J* = 14.5 Hz), 6.88 (t, 1H, *J* = 12.5Hz), 3.72 (d, 2H, *J* = 12.5Hz), 2.34 (s, 3H), 2.32 (s, 3H). High-resolution mass spectrometry (HRMS, electrospray ionisation, ESI) *m/z* $[M+H]^+$ cacld. 338.1154, found 339.1183.

Compound **2**: To a solution of compound **3** (50 mg, 0.147 mmol) in methanol (6 mL) potassium carbonate (51 mg, 0.368 mmol) was added, and the reaction mixture was stirred at room temperature for 15 min. After completing the reaction, the solvent was removed in *vacuo*, diluted with dichloromethane (DCM, 50 mL), the organic layer was washed with water (2 × 20 mL), and then dried over anhydrous magnesium sulphate ($MgSO_4$) to evaporate the solvent. This was followed by column purification (1:1, hexane: ethyl acetate) to obtain a yellow liquid (26 mg, 70%). $^1$H NMR (500 MHz, $CDCl_3$) δ. 7.09 (d, 2H, *J* = 8.5Hz), 6.94 (d, 2H, *J* = 8.5Hz), 6.8 (d, 2H, *J* = 8.5Hz), 6.68 (d, 2H, *J* = 8.5Hz), 5.8 (t, 1H, *J* = 7.5Hz), 5.43 (br s, 1H), 5.36 (br s, 1H), 4.23 (s, 2H), 3.67 (br s, 1H), 3.23 (d, 2H, *J* = 7.5Hz). HRMS (ESI) *m/z* $[M+H]^+$ cacld. 256.1099, found 257.1145.

Compound **3**: To a solution of compound **1** (50 mg, 0.148 mmol) in ethanol (10 mL), sodium borohydride ($NaBH_4$, 7 mg, 0.185 mmol) was added at 0°C, and the reaction mixture was stirred at room temperature for 1 h. After completing the reaction, the solvent was removed in *vacuo*, diluted with water (10 mL), and the compound was extracted with DCM (2 × 15 mL). The

separated organic layer was dried over anhydrous $MgSO_4$ to evaporate the solvent, followed by column purification (2:1, hexane: ethyl acetate) to yield a colourless liquid (37 mg, 78%). $^1$H NMR (500 MHz, $CDCl_3$) δ. 7.30 (d, 2H, *J* = 14Hz), 7.18-7.12 (m, 4H), 7.02 (d, 2H, *J* = 14Hz), 5.93 (t, 1H, *J* = 12.5Hz), 4.36 (s, 2H), 3.37 (d, 2H, *J* = 12.5Hz), 2.34 (s, 3H), 2.31 (s,3H). HRMS (ESI) *m/z* $[M+H]^+$ cacld 340.13107, found 341.1343.

Compound **4**: To a solution of compound **2** (40 mg, 0.156 mmol) in dimethylformamide (DMF), potassium carbonate (54 mg, 0.39 mmol) and methyl iodide (29 µL, 0.468 mmol) were added. Then, the reaction mixture was stirred at room temperature for 12 h, diluted with water (15 mL), the compound was extracted with DCM (2 × 30 mL), separated, and then the organic layer was dried over anhydrous $MgSO_4$, to evaporate the solvent. This was followed by column purification (2:1, hexane:ethyl acetate) to yield a colourless liquid (37 mg, 86%). $^1$H NMR (500 MHz, $CDCl_3$) δ. 7.21 (d, 2H, *J* = 8.5Hz), 7.06 (d, 2H, *J* = 8.5Hz), 6.93 (d, 2H, *J* = 8.5Hz), 6.83 (d, 2H, *J* = 8.5Hz), 5.86 (t, 1H, *J* = 7.5Hz), 4.35 (s, 2H), 3.83 (s, 3H), 3.78 (s, 3H), 3.61 (br s, 1H), 3.32 (d, 2H, *J* = 7.5Hz). HRMS (ESI) *m/z* $[M+H]^+$ cacld. 284.14124, found 285.1465.

Compound **5**: To a solution of compound **2** (40 mg, 0.156 mmol) in DMF, 50% sodium hydride (NaH, 30 mg, 0.624 mmol) was added at 0°C, with stirring for 10 min. Then, methyl iodide (48 µL, 0.78 mmol) was added; the reaction mixture was stirred at room temperature for 1 h, and after the reaction was completed, it was monitored using thin layer chromatography (TLC). The reaction mixture was quenched with water (10 mL), the compound was extracted with DCM (2 × 30 mL), separated, and then the organic layer was dried over anhydrous $MgSO_4$, to evaporate the solvent, followed by column purification (3:1, hexane: ethyl acetate) to yield a pale yellow liquid (40 mg, 70%). $^1$H NMR (500 MHz, $CDCl_3$) δ. 7.24 (d, 2H, *J* = 15Hz), 7.10 (d, 2H, *J* = 15Hz), 6.94 (d, 2H, *J* = 15Hz), 6.85 (d, 2H, *J* = 15Hz), 5.90 (t, 1H, *J* = 12.5Hz), 5.29 (br s, 1H), 4.17 (s,

2H), 3.85 (s, 3H), 3.82 (s, 3H), 3.34 (d, 2H, $J$ = 12.5Hz), 3.37 (s, 3H). HRMS (ESI) *m/z* $[M+H]^+$ cacld. 298.15689, found 299.1601.

Compound **6**: To a solution of compound **2** (40 mg, 0.156 mmol) in DMF, 50% NaH (14 mg, 0.296 mmol) was added at 0°C with stirring for 10 min. Then, benzyl chloride (34 µL, 0.296 mmol) was added, the reaction mixture was stirred at room temperature for 1 h, and after completing the reaction, which was monitored usingTLC, the reaction mixture was quenched with water (10 mL). The compound was then extracted with DCM (2 × 30 mL), separated, and the organic layer was dried over anhydrous $MgSO_4$, to evaporate the solvent followed by column purification (3:1, hexane:ethyl acetate) to yield a colourless liquid (40 mg, 66%). %). $^1H$ NMR (500 MHz, $CDCl_3$) δ. 7.49-7.34 (m, 10H), 7.23 (d, 2H, $J$ = 14.5Hz), 7.09 (d, 2H, $J$ = 14.5Hz), 7.03 (d, 2H, $J$ = 14.5Hz), 6.93 (d, 2H, $J$ = 14.5Hz), 5.89 (t, 1H, $J$ = 12.5Hz), 5.1 (s, 2H), 5.07 (s, 2H), 4.37 (s, 2H), 3.35 (d, 2H, $J$ = 12.5Hz). HRMS (ESI) *m/z* $[M+NH_4]^+$ cacld. 436.54152, found 454.2336.

Compound **7**: To a solution of compound **2** (40 mg, 0.156 mmol) in DMF, 50% NaH (30 mg, 0.624 mmol) was added at 0°C with stirring for 10 min. Then benzyl chloride (63 µL, 0.546 mmol) was added, the reaction mixture was stirred at room temperature for 1 h, and completion of the reaction, which was monitored using TLC, the reaction mixture was quenched with water (10 mL). The compound was extracted with DCM (2 × 30 mL), separated, and the organic layer was dried over anhydrous $MgSO_4$, to evaporate the solvent, followed by column purification (3:1, hexane:ethyl acetate) to yield a colourless liquid (70 mg, 85%).$^1H$ NMR (500 MHz, $CDCl_3$) δ. 7.47-7.26 (m, 15H), 7.23 (d, 2H, $J$ = 8.5Hz), 7.09 (d, 2H, $J$ = 8.5Hz), 7.03 (d, 2H, $J$ = 8.5Hz), 6.93 (d, 2H, $J$ = 8.5Hz), 5.91 (t, 1H, $J$ = 7.5Hz), 5.09 (s, 2H), 5.05 (s, 2H), 4.55 (s, 2H), 4.24 (s, 2H), 3.37 (d, 2H, $J$ = 7.5Hz). HRMS (ESI) *m/z* $[M+NH_4]^+$ cacld. 526.25079, found 544.2787.

Compound **8**: To a solution of compound **1** (50 mg, 0.148 mmol) in ethyl acetate (15 mL), 10% Pd/C was added, and the reaction mixture was stirred under $H_2$ atmosphere at room temperature for 4 h. After completion of the reaction, the catalyst was removed by filtration through a celite pad. The evaporation of solvent, followed by column purification (2:1, hexane: ethyl acetate) yielded a colourless liquid (40 mg, 80%). $^1$H NMR (500 MHz, $CDCl_3$) δ. 9.68 (s, 1H), 7.24 (d, 2H, *J* = 14.5Hz), 7.17 (dd, 4H, *J* = 10, 14.5Hz), 7.02 (d, 2H, *J* = 14.5Hz), 3.55 (t, 1H, *J* = 11Hz), 2.66-2.53, (m, 2H), 2.49-2.37 (m, 1H), 2.34 (s, 3H), 2.32 (s, 3H), 2.11-1.99 (m, 1H). HRMS (ESI) *m/z* $[M+Na]^+$ cacld. 340.13107, found 363.1249.

Compound **9**: To a solution of compound **10** (70 mg, 0.205 mmol) in methanol (10 mL), potassium carbonate (71 mg, 0.513 mmol) was added, and the reaction mixture was stirred at room temperature for 15 min. After completion of the reaction, the solvent was removed in *vacuo*, the residue was diluted with DCM (50 mL), the organic layer was washed with water (2 × 10 mL), and dried over anhydrous $MgSO_4$, to evaporate the solvent. This was followed by column purification (1:1 hexane: ethyl acetate) to yield a colourless liquid (40 mg, 76%). $^1$H NMR (500 MHz, $CDCl_3$) δ. 7.03 (d, 2H, *J* = 8.5Hz), 6.91 (d, 2H, *J* = 8.5Hz), 6.76 (d, 2H, *J* = 8.5Hz), 6.66 (d, 2H, *J* = 8.5Hz), 3.60-3.58 (m, 2H), 2.65-2.59 (m, 1H), 2.43-2.37 (m, 1H), 2.34-2.28 (m, 2H), 2.08-2.02 (m, 1H), 1.78-1.71 (m, 1H). HRMS (ESI) *m/z* $[M+H]^+$ cacld. 258.12559, found 259.1154.

Compound **10**: To a solution of compound **3** (30 mg, 0.088 mmol) in ethyl acetate (15 mL), 10% Pd/C was added, and the reaction mixture was stirred at room temperature for 4 h. After completion of the reaction, the catalyst was removed by filtration through a celite pad. The evaporation of the solvent was followed by column purification (2:1 hexane: ethyl acetate), which yielded a colourless liquid (23 mg, 76%). $^1$H NMR (500 MHz, $CDCl_3$) δ. 7.29 (d, 2H, *J* =

14.5Hz), 7.15 (t, 4H, *J* = 13.5Hz), 6.99 (d, 2H, *J* = 14.5Hz), 3.76 (brs, 2H), 3.63 (br s, 1H), 2.89-2.79 (m, 1H), 2.60-2.45 (m, 2H), 2.31 (s, 3H), 2.27 (s, 3H), 2.14-2.02 (m, 1H), 1.96-1.86 (m, 1H). HRMS (ESI) *m/z* $[M+H]^+$ cacld. 342.14672, found 343.1454.

***General procedure for the synthesis of the compounds 11-16:*** Compounds **11-16** were prepared using the following Heck reaction condition starting from phenyl halide moieties with substituents (2.0 mmol) and allylbenzene moieties with substituents (2.0 mmol). The phenyl halide (2.0 mmol) and allylbenzene (2.0 mmol) along with triphenylphosphine (105 mg, 0.4 mmol), $Pd(OAc)_2$ (44.9 mg, 0.2 mmol), and tributylamine (451 μL, 1.9 mmol) were mixed in a 25-mL round bottom flask, and the reaction mixture was stirred for 2 h at 45°C or for 2 days at an ambient temperature under an argon atmosphere. The product was purified using flash silica gel chromatography using hexane and ethyl acetate (3:1, v/v) as the mobile phase.

Compound **13** ([E]-2-methoxy-4-(3-(4-methoxyphenyl) prop-1-en-1-yl) phenol, MMPP): $^1$H-NMR (500 MHz, $CDCl_3$) δ 7.32 (d, 2H, *J* = 8.0 Hz), 6.88 (d, 1H, *J* = 9.0 Hz), 6.86 (d, 2H, *J* = 9.0 Hz), 6.76 (d, 1H, *J* = 8.0 Hz), 6.75 (s, 1H), 6.40 (d, 1H, *J* = 16.0 Hz), 6.21 (dt, 1H, *J* = 16.0 Hz, *J* = 6.5 Hz), 5.54 (s, 1H), 3.89 (s, 3H), 3.82 (s, 3H), 3.48 (d, 2H, 7.0 Hz). HRMS (ESI) *m/z* $[M+H]^+$ cacld. 271.1329, found 271.1332. The MMPP structure is shown in Fig. 1a.

Compound **16**: $^1$H-NMR (500 MHz, $CDCl_3$) δ 6.88-6.91 (m, 4H), 6.76-6.78 (m, 2H), 6.38 (d, 1H, *J* = 16.0 Hz), 6.20 (dt, 1H, *J* = 16.0 Hz, *J* = 6.5 Hz), 5.30 (br s, 1H), 3.90 (s, 3H), 3.89 (s, 3H), 3.48 (d, 2H, 6.5 Hz). HRMS (ESI) *m/z* $[M+H]^+$ cacld. 301.1440, found 301.1452.

**RAW264.7 Cell culture**

The murine macrophage-like RAW264.7 cell line was obtained from the American Type Culture Collection (ATCC, Manassas, VA, USA), were cultured in Dulbecco's modified Eagle’s medium

(DMEM, Gibco-BRL) with 10% heat-inactivated foetal bovine serum (FBS) and penicillin/streptomycin (100 U/mL) at 37°C under a humidified atmosphere containing 5% $CO_2$ inside a $CO_2$ incubator.

**Human synoviocytes culture and ethics statement**

The Clinical Research Ethics Committee of the College of Medicine, Soonchunhyang University Medical Center, approved the study protocol and the use of human tissues. Informed consent was obtained from all patients. Patients with rheumatoid arthritis (RA) were diagnosed according to the 1987 Revised Criteria of the American College of Rheumatology. The synovial tissue samples were obtained from male and female patients (two each) with long-standing RA, aged 65 ± 21.3 years (mean ± SD) with a mean disease duration ≥10 years at the time of a total knee joint replacement. The human fibroblast-like synoviocytes (FLSs) were cultured as previously described[43]. In brief, the FLSs were propagated in culture dishes (Nalge Nunc International, Rochester, NY, USA) in DMEM supplemented with 20% heat-inactivated FBS (Gibco-BRL, Grand Island, NY, USA) and penicillin/streptomycin (50 U/mL) at 37°C under a humidified atmosphere containing 5% $CO_2$ inside a $CO_2$ incubator. The medium was changed every 3 days, and the cells were used between the fifth and the tenth passages.

**Animals and ethics statement**

Male C57BL/7 mice (7-week-old) were obtained from Taconic Korea (Daehan Biolink Co., Ltd., Umsung, Chungbuk, Korea). The animals were housed under specific pathogen-free conditions with three to four animals per cage on dust free plant fibre bedding and maintained at 23 ± 2°C with a controlled 12-h light/dark cycle. In addition, drinking water and rodent chow diet were provided *ad libitum* throughout the experiment. All the animal experiments were conducted in accordance with the principles and procedures outlined in the National Institute of Health (NIH)

Guide for the Care and Use of Laboratory Animals. The protocol for the animal experiments was approved by the Animal Ethics Committee at Chungbuk National University.

**CAIA induction and dosing**

The mice were administered 5 mg collagen II antibody cocktail (CII-Ab, Arthrogen-CIA Arthritogenic Monoclonal Antibody, # 53010: Chondrex Inc., Redmond, WA, USA) intravenously on day 1 and then challenged with 50 μg lipopolysaccharide (LPS) intraperitoneally (i.p.) on day 3. The disease onset occurred on day 4, and the mice were visually examined daily for the appearance of arthritis in the peripheral joints. The severity of arthritis was graded on a scale of 0–4 for each paw in a blinded fashion: 0, no evidence of erythema or swelling; 1, erythema and mild swelling confined to the midfoot (tarsals) or ankle joint; 2, erythema and mild swelling extending from the ankle to the midfoot; 3, erythema and moderate swelling extending from the ankle to the metatarsal joints; and 4, erythema and severe swelling encompassing the ankle, foot and digits. The scores for each of the four paws were added to obtain a final score, with a maximal severity score of 16, presented as the mean ± standard error of the mean (SEM). The mice were treated i.p. with the vehicle (0.05% dimethyl sulphoxide [DMSO] in normal saline), MMPP (5 mg/kg), or indomethacin (5 mg/kg, positive control), daily from day 0 to the end of the experiment (21 days). On day 21, the mice were anaesthetised and placed on a radiographic box at a distance of 90 cm from an X-ray source. Then, the radiographic analysis of the arthritic hind paws was performed using an X-ray machine (BLD-150RK, Hradec Králové, Czech Republic) with a 40 KW exposition for 0.01 s.

**Histological analysis**

The animals were euthanized using $CO_2$ gas inhalation, the liver and paw tissue was collected,

and histological analyses were performed as previously described[33]. In brief, the ankle joints and liver tissues were fixed in 4% formalin, embedded in paraffin, and serially cut into 4-μm thick sections, which were stained with haematoxylin and eosin (H&E). The immunohistochemical staining was carried out using antibodies against cyclooxygenase (COX)-2 and inducible nitric oxide synthase (iNOS, Cayman Chemical, Ann Arbor, MI, USA).

**CBC tests**

The whole blood samples were collected from mice for complete blood count (CBC). The absolute neutrophils and monocytes were counted using a high-volume haematology analyser (ADVIA 2120i, Siemens AG, Henkestrasse, Erlangen, Germany).

**Cell viability assays**

Cells were plated in 24-well plates ($5 \times 10^4$ cells/well) and subconfluent cells were treated with MMPP (1, 2, and 4 μg/mL) for 24 h. After treatment, cells were trypsinised and pelleted by centrifugation for 5 min at 1,500 rpm, resuspended in 10 mL phosphate-buffered saline (PBS), and 0.1 mL 0.2% trypan blue was added to the cell suspension in each of the solutions (0.9 mL each). Subsequently, a drop of the suspension was placed in a Neubauer chamber, and the living cells were counted. Cells that showed signs of staining were considered dead, whereas those that excluded trypan blue were considered viable. Each assay was carried out in triplicate.

**NO and hydrogen peroxide ($H_2O_2$) measurements**

Cells were plated at a density of $1 \times 10^4$ cells/well in 96-well culture plates and subsequently incubated with or without LPS (1 μg/mL) and TNF-α (10 ng/mL) in the absence or presence of various concentrations of MMPP for 24 h. The NO production was directly measured by

detecting the accumulated nitrite in the culture medium using an NO detection kit (iNtRON Biotechnology Inc., Seongnam, Korea) according to the manufacturer's instructions. The hydrogen peroxide ($H_2O_2$) produced by cells in the culture medium was measured using a fluorometric assay kit (Cell Biolabs Inc., San Diego, CA, USA).

### Prostaglandin $E_2$ ($PGE_2$) and cytokine measurements

Prostaglandin $E_2$ ($PGE_2$) and cytokine measurements were performed in cultured RAW264.7 cells and CAIA-induced animals. For the measurement in cultured cells, they were plated at a density of $1 \times 10^4$ cells/well in a 96-well culture plate and subsequently incubated with or without LPS (1 μg/mL) and TNF-α (10 ng/mL) in the absence or presence of various concentrations of MMPP for 24 h. The culture medium was collected and the $PGE_2$, tumour necrosis factor (TNF)-α, interleukin (IL)-1β, and IL-6 levels were analysed using enzyme-linked immunosorbent assay (ELISA) kits (R&D Systems Inc., Minneapolis, MI, USA) according to the manufacturer's instructions. For the measurements in mouse tissue, the ankle joint tissues were isolated from normal and CAIA mice treated with MMPP (5 mg/kg), indomethacin (5 mg/kg), or vehicle and parts of the tissues were rinsed with ice-cold PBS, homogenised on ice, and centrifuged. The supernatants were collected and measured using the same protocol described above.

### Reporter gene assay

The cells were plated at a density of $1 \times 10^5$ cells/well in a 24-well culture plate and transiently transfected with STAT3-luciferase reporter (Affymetrix Inc., Santa Clara, CA, USA) or phosphorylated-nuclear factor kappa-light-chain-enhancer of activated B cells (p-NF-κB)-luciferase reporter (Stratagene, Cedar Cree, CA, USA) using Lipofectamine LTX and PLUS

(Invitrogen) in OPTI-MEM media (Invitrogen, Carlsbad, CA, USA) according to the manufacturer's instruction. The transfected cells were treated with LPS (1 μg/mL) in the absence or presence of various concentrations of MMPP for 8 h. The reporter gene activity was assayed using the luciferase assay kit (Promega Co., Madison, WI, USA) and measured using a luminescence counter (Wallac Victor2 1420, PerkinElmer Inc., Waltham, MA, USA).

**Western blot analysis**

The whole cell lysates, cytosolic extract, and nuclear extract of cultured cells and ankle joint tissues were obtained, separated using sodium dodecyl sulphate-polyacrylamide gel electrophoresis (SDS-PAGE), and a western blot analysis was performed as described previously[33]. The proteins were transferred to polyvinylidene fluoride (PVDF) membranes, which were blocked with 5% skim milk in Tris-buffered saline plus Tween (TBS/T) for 2.5 h at room temperature. The protein transfer membranes were probed with the following primary antibodies: mouse polyclonal antibodies against p50, p-IκB, histone H1, and β-actin (1:500), mouse monoclonal antibodies against p-STAT3 and STAT3 (1:500), rabbit polyclonal antibodies against p65, p-IKKα, IKKα, p-IKKβ, IKKβ, and IκB (1:500, all Santa Cruz Biotechnology Inc. Santa Cruz, CA, USA), iNOS and COX-2 (1:1000, Cayman Chemical, Ann Arbor, MI, USA). The protein expression was visualised using a chemiluminescence reagent (Amersham Pharmacia Biotech, Inc., Buckinghamshire, UK), and detected using a digital chemiluminescence imaging system equipped with a charge-coupled device (CCD) camera (Fusion-FX, Fisher BioTech Ltd., Wembley, Australia).

**DNA-binding activity assay by electromobility shift assay (EMSA)**

The DNA-binding activities of STAT3 and NF-κB in the cultured cells and ankle joint tissues of

CAIA mice were determined using an electromobility shift assay (EMSA) as described previously[33]. In brief, cells were treated with MMPP or DMSO. After incubation for 24 h, the cells were washed thrice with ice-cold PBS and the nuclear extracts were prepared for EMSA. For the measurement in mouse tissue, the ankle joint tissues were isolated from normal and CAIA mice treated with MMPP (5 mg/kg), indomethacin (5 mg/kg), or vehicle, and parts of the tissues were rinsed with ice-cold PBS, homogenised in ice-cold buffer A (10 mM potassium chloride [KCl], 0.2 mM ethylenediaminetetraacetic acid [EDTA], 1.5 mM magnesium chloride [$MgCl_2$], 0.5 mL dithiothreitol [DTT], and 0.2 mM phenylmethanesulfonyl fluoride [PMSF]) and centrifuged for 5 min at 14,000 × *g*. The residual pellet was resuspended in 100 µL buffer C (20 mM 4-(2-hydroxyethyl)-1-piperazineethanesulfonic acid [HEPES], 420 mM sodium chloride (NaCl), 1.5 mM MgCl2, 20% glycerol, 0.2 mM EDTA, 0.5 mM DTT and 0.2 mM PMSF). After incubation at 4°C for 20 min, the lysate was centrifuged for 6 min at 14,000 × *g* and the supernatants (nuclear extract) were collected, and EMSA was carried out as described above.

**Statistical analysis**

The statistical analysis was carried out using the statistical package for the social sciences (SPSS) version 18.0. All error bars reported are standard error of the mean (SEM) unless otherwise indicated. Pairwise comparisons were performed using a one-way Student’s *t*-test. Multiple comparisons of means were performed using a one-way analysis of variance (ANOVA) followed by Tukey’s multiple comparison tests. The differences between groups were considered significant at *P*-values < 0.05

## REFERENCES


1 Ban, J. O. *et al.* Anti-arthritis effects of (E)-2,4-bis(p-hydroxyphenyl)-2-butenal are mediated by inhibition of the STAT3 pathway. *British journal of pharmacology* **171**, 2900-2912, doi:10.1111/bph.12619 (2014).

2 Lee, Y. J. *et al.* Inhibitory effect of a tyrosine-fructose Maillard reaction product, 2,4-bis(p-hydroxyphenyl)-2-butenal on amyloid-beta generation and inflammatory reactions via inhibition of NF-kappaB and STAT3 activation in cultured astrocytes and microglial BV-2 cells. *Journal of neuroinflammation* **8**, 132, doi:10.1186/1742-2094-8-132 (2011).

3 Jin, P. *et al.* Anti-inflammatory and anti-amyloidogenic effects of a small molecule, 2,4-bis(p-hydroxyphenyl)-2-butenal in Tg2576 Alzheimer's disease mice model. *Journal of neuroinflammation* **10**, 2, doi:10.1186/1742-2094-10-2 (2013).

4 Kim, J. A. *et al.* Inhibitory effect of a 2,4-bis(4-hydroxyphenyl)-2-butenal diacetate on neuro-inflammatory reactions via inhibition of STAT1 and STAT3 activation in cultured astrocytes and microglial BV-2 cells. *Neuropharmacology* **79**, 476-487, doi:10.1016/j.neuropharm.2013.06.032 (2014).

5 Kollipara, P. S., Jeong, H. S., Han, S. B. & Hong, J. T. (E)-2,4-bis(p-hydroxyphenyl)-2-butenal has an antiproliferative effect on NSCLC cells induced by p38 MAPK-mediated suppression of NF-kappaB and up-regulation of TNFRSF10B (DR5). *British journal of pharmacology* **168**, 1471-1484, doi:10.1111/bph.12024 (2013).

6 Kim, M. S. *et al.* 2,4-bis (p-hydroxyphenyl)-2-butenal (HPB242) induces apoptosis via modulating E7 expression and inhibition of PI3K/Akt pathway in SiHa human cervical cancer cells. *Nutrition and cancer* **64**, 1236-1244, doi:10.1080/01635581.2012.718405 (2012).

7 Lee, U. S. *et al.* Growth Inhibitory Effect of (E)-2,4-bis(p-hydroxyphenyl)-2-Butenal Diacetate through Induction of Apoptotic Cell Death by Increasing DR3 Expression in Human Lung Cancer Cells. *Biomolecules & therapeutics* **20**, 538-543, doi:10.4062/biomolther.2012.20.6.538 (2012).

8 Ban, J. O. *et al.* (E)-2,4-Bis(p-hydroxyphenyl)-2-butenal inhibits tumor growth via suppression of NF-kappaB and induction of death receptor 6. *Apoptosis : an international journal on programmed cell death* **19**, 165-178, doi:10.1007/s10495-013-0903-x (2014).

9 Cho, S. H. *et al.* (E)-2,4-Bis(p-hydroxyphenyl)-2-butenal enhanced TRAIL-induced apoptosis in ovarian cancer cells through downregulation of NF-kappaB/STAT3 pathway. *Archives of pharmacal research* **37**, 652-661, doi:10.1007/s12272-013-0326-9 (2014).

10 Chae, J. I., Lee, R., Cho, J., Hong, J. & Shim, J. H. Specificity protein 1 is a novel target of 2,4-bis (p-hydroxyphenyl)-2-butenal for the suppression of human oral squamous cell carcinoma cell growth. *Journal of biomedical science* **21**, 4, doi:10.1186/1423-0127-21-4 (2014).

11 Nah, S. S. *et al.* Epidermal growth factor increases prostaglandin E2 production via ERK1/2 MAPK and NF-kappaB pathway in fibroblast like synoviocytes from patients with rheumatoid arthritis. *Rheumatol Int* **30**, 443-449, doi:10.1007/s00296-009-0976-6 (2010).

## Supplementary Results

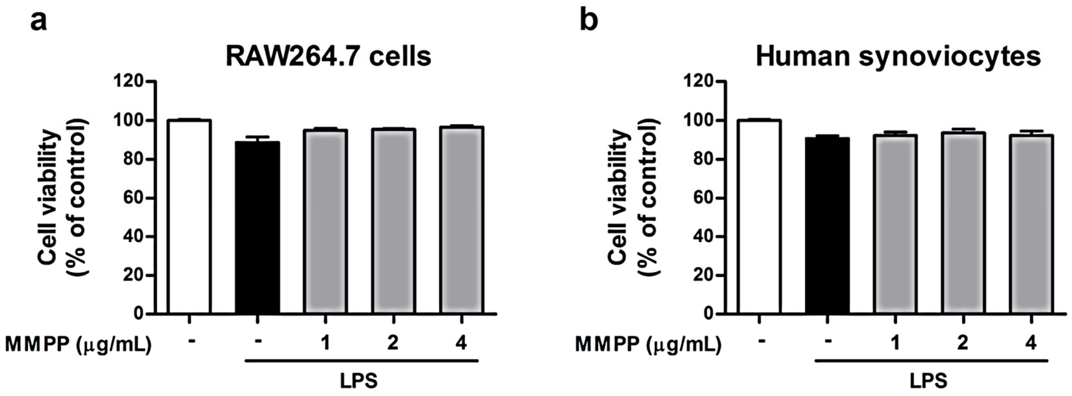


**Supplementary Figure S1. Effect of (E)-2-methoxy-4-(3-(4-methoxyphenyl) prop-1-en-1-yl) phenol (MMPP) on viability of RAW264.7 cells and human synoviocytes** (**a**) Murine macrophage-like RAW264.7 cells and (**b**) human synoviocytes were pre-treated with MMPP (1, 2, and 4 µg/mL) for 24 h, and then stimulated with lipopolysaccharide (LPS, 1 µg/mL) for 24 h. The cell viability was examined by cell counting (trypan blue staining) as described in Supplementary Methods. All data shown as mean ± standard error of the mean (SEM) of three independent experiments.

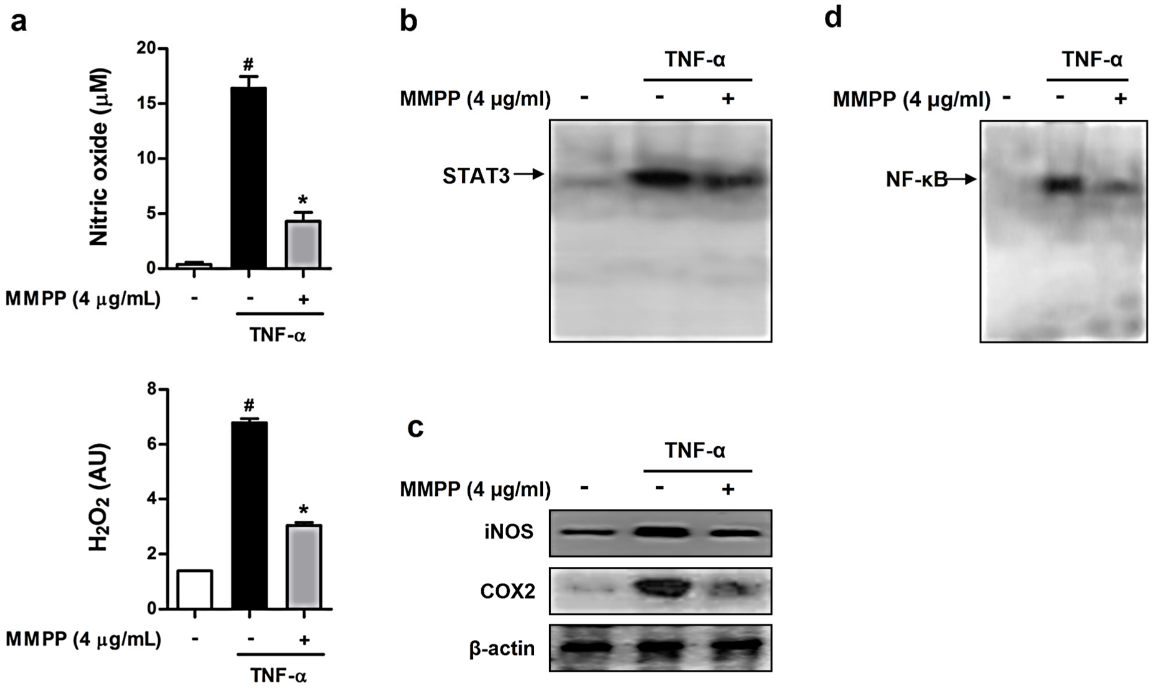


**Supplementary Figure S2. Effect of (E)-2-methoxy-4-(3-(4-methoxyphenyl) prop-1-en-1-yl) phenol (MMPP) on tumour necrosis factor (TNF)-α-induced nitric oxide (NO) and hydrogen peroxide ($H_2O_2$) production and signal transducer and activator of transcription 3 (STAT3) and NF-κB DNA binding activity and pro-inflammatory protein expression in RAW264.7 cells.** (**a–c**) Murine macrophage-like cell line RAW264.7 cells were pre-treated with MMPP (4 μg/mL) for 24 h and then stimulated with TNF-α (10 ng/mL) for 24 h. (**a**) Levels of NO and $H_2O_2$ were determined (n = 6, data are mean ± standard error of the mean, SEM, $^{\#}P <$ 0.05 *vs.* intact control and $^{*}P < 0.05$ *vs.* TNF-α-treated control using Student's *t*-test.). (**b**) DNA-binding activity of STAT3 and NF-κB was determined using electrophoretic mobility shift assay (EMSA) using nuclear extracts. (**c**) Cells were lysed and analysed using western blotting with antibodies against iNOS and COX2, using β-actin as a loading control. iNOS, inducible nitric oxide synthase; COX, cyclooxygenase.

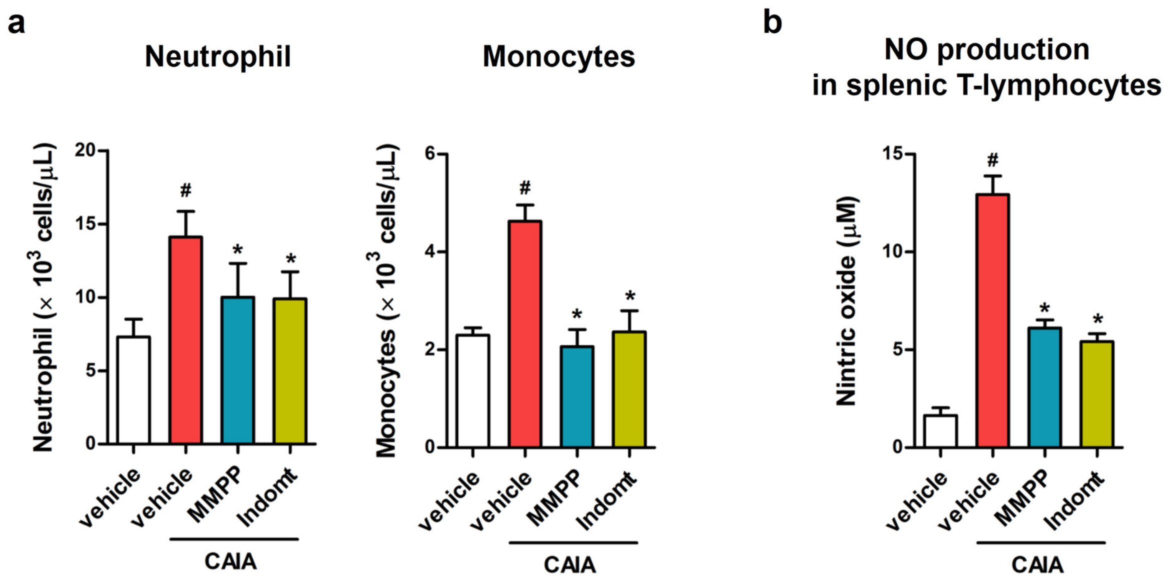


**Supplementary Figure S3. Effect of Effect of (E)-2-methoxy-4-(3-(4-methoxyphenyl) prop-1-en-1-yl) phenol (MMPP) on circulating inflammatory cell population and splenic T-lymphocytes nitric oxide (NO) production in collagen antibody-induced arthritis (CAIA) mice** (**a–b**) C57BL/6 mice were administered 5 mg collagen II antibody cocktail intravenously and challenged with 50 µg of lipopolysaccharide (LPS) intraperitoneal. Vehicle (normal saline containing 0.05% dimethyl sulphoxide (DMSO)-treated mice without antibody-LPS injection were used as normal control (white bars). Vehicle-treated mice with antibody-LPS injection were used as CAIA-control (red bars). Mice were treated with 5 mg/kg of MMPP (blue bars) or 5 mg/kg of indomethacin (indomt, yellow bars) daily for 21 days. (**a**) Number of circulating neutrophils and monocytes in mouse blood was analysed using high-volume haematology analyser. (**b**) Levels of NO production in splenic T-lymphocytes were determined. Data are mean ± SEM (n = 10). $^{\#}P < 0.05$ *vs.* normal control and $^{*}P < 0.05$ *vs.* CAIA-control as determined by paired *t*-test.

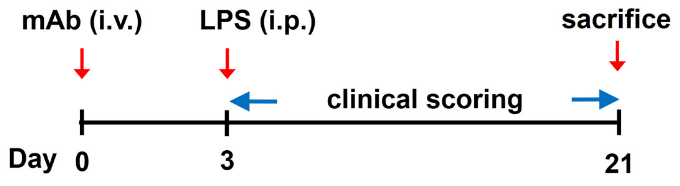


**Supplementary Figure S4. Schematic diagram of induction of CAIA in mouse.** C57BL/6 mice were administered 5 mg collagen II antibody cocktail intravenously (i.v.) on day 0, then challenged with 50 µg of lipopolysaccharide (LPS) intraperitoneal (i.p.) on day 3. Mice were treated i.p. with 0.05% DMSO in normal saline (CAIA-control), 5 mg/kg MMPP, or 5 mg/kg indomethacin daily during entire experimental period. On day 21, severity of RA in hind paws was examined by photo-imaging and radiographic analysis.

**Supplementary Table S1. Effect of synthetic BHPB analogues on signal transducer and activator of transcription 3 (STAT3) DNA-binding activity and nitric oxide (NO) generation in lipopolysaccharide (LPS)-stimulated RAW264.7 cells**

| No. | Compound name | $IC_{50}$ value on NO generation (μg/mL) | $IC_{50}$ value on STAT3 DNA binding activity (μg/mL) |
|---|---|---|---|
| | (E)-2,4-bis(4-hydroxyphenyl)but-2-enal (BHPB) | 6.42 | 5.91 |
| **1** | (E)-2,4-bis(4-hydroxyphenyl)but-2-enal diacetate | 2.97 | 2.47 |
| **2** | (E)-4,4'-(4-hydroxybut-2-ene-1,3-diyl) diphenol | 4.61 | 4.65 |
| **3** | (E)-4,4'-(4-hydroxybut-2-ene-1,3-diyl)diphenol diacetate | 4.54 | 4.36 |
| **4** | (E)-2,4-bis(4-methoxyphenyl)but-2-en-1-ol | 6.41 | 5.87 |
| **5** | (E)-4,4'-(4-methoxybut-2-ene-1,3-diyl)bis(methoxybenzene) | 6.50 | 3.46 |
| **6** | (E)-4-(4-(benzyloxy)-3-(4-(benzyloxy)phenyl)but-2-en-1-yl)phenol | 2.61 | 2.24 |
| **7** | (E)-4,4'-(4-(benzyloxy)but-2-ene-1,3-diyl)bis((benzyloxy)benzene) | 3.12 | 4.10 |
| **8** | (E)-2,4-bis(4-hydroxyphenyl)butanal diacetate | 2.97 | 5.80 |
| **9** | 4,4'-(4-hydroxybutane-1,3-diyl)diphenol | 2.72 | 3.10 |
| **10** | 4,4'-(4-hydroxybutane-1,3-diyl)diphenol diacetate | 4.54 | 3.44 |
| **11** | (E)-4-(3-(4-methoxyphenyl)prop-1-en-1-yl)phenol | 2.97 | 2.15 |
| **12** | (E)-4-(3-(4-hydroxyphenyl)allyl)-2-methoxyphenol | 2.29 | 2.10 |
| **13** | (E)-2-methoxy-4-(3-(4-methoxyphenyl)prop-1-en-1-yl)phenol (MMPP) | 1.79 | 2.05 |
| **14** | (E)-4,4'-(prop-1-ene-1,3-diyl)bis(2-methoxyphenol) | 1.84 | 2.23 |
| **15** | (E)-1,3-dimethoxy-5-(3-(4-methoxyphenyl)prop-1-en-1-yl)benzene | 3.17 | 4.60 |
| **16** | (E)-4-(3-(3,5-dimethoxyphenyl)allyl)-2-methoxyphenol | 3.22 | 3.10 |
| | Curcumin | 5.21 | 4.01 |
| | Tofacitinib | 5.53 | 4.12 |
